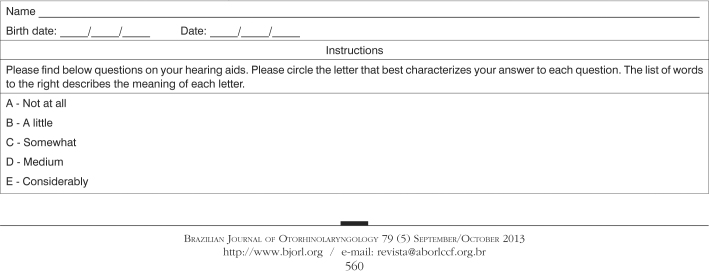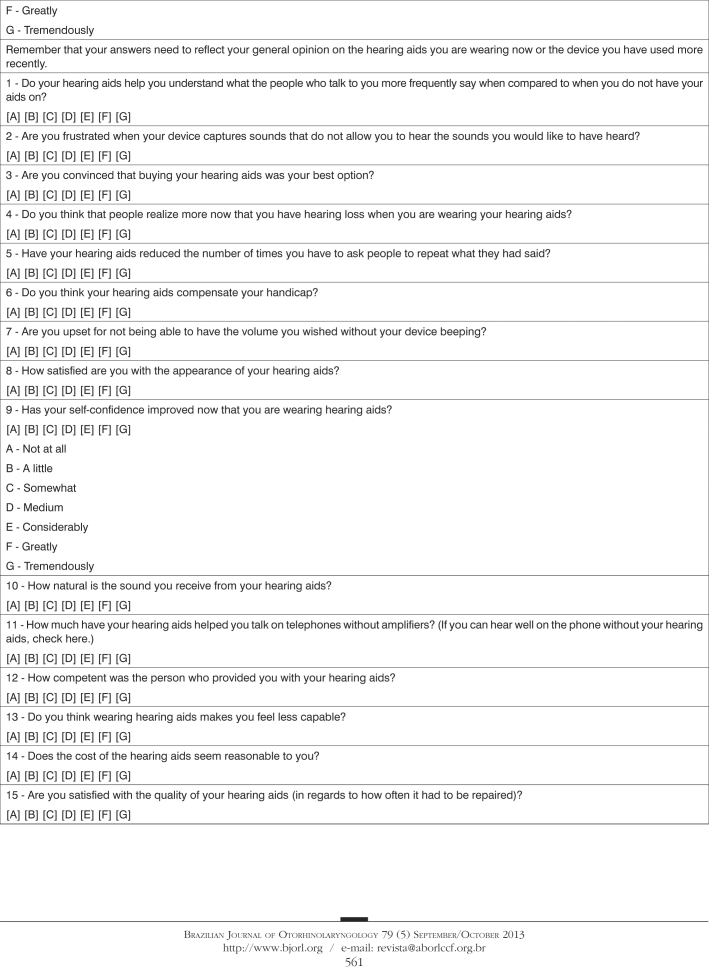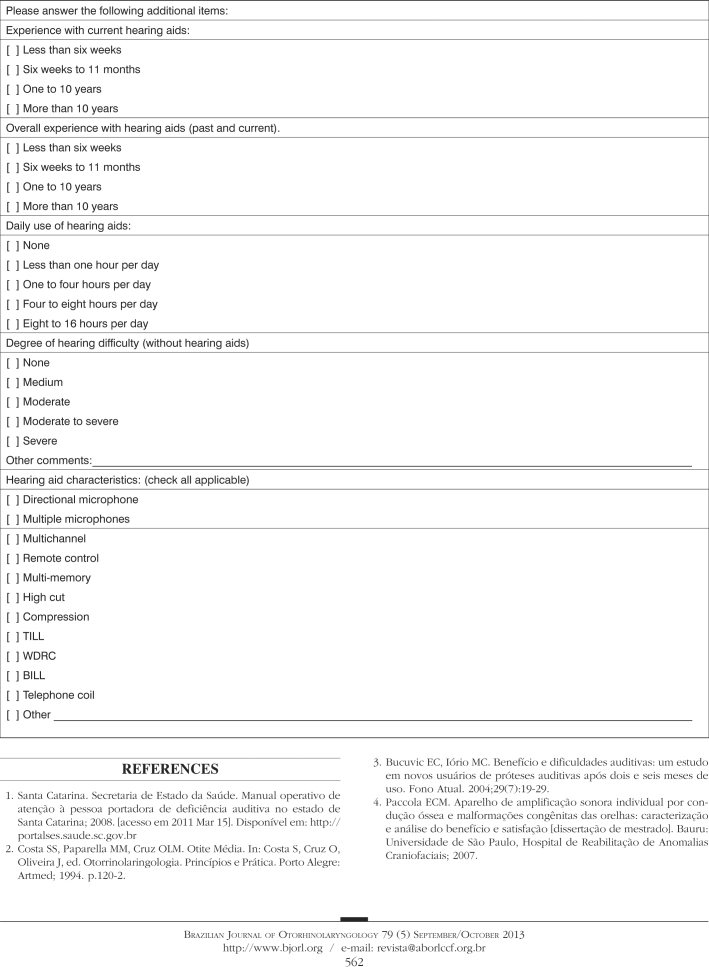# Degree of satisfaction of patients fitted with hearing aids at a high complexity service

**DOI:** 10.5935/1808-8694.20130100

**Published:** 2015-10-08

**Authors:** Sabrina Freiberger Dell'Antônia, Claudio Marcio Yudi Ikino, Waldir Carreirão Filho

**Affiliations:** a Medical Student (Medical Student, Federal University of Santa Catarina).; b PhD in Sciences, School of Medicine, University of São Paulo (Adjunct Professor, Surgery Department, Federal University of Santa Catarina); c MSc in Otorhinolaryngology, Pontifical Catholic University of Rio de Janeiro (Adjunct Professor, Surgery Department, Federal University of Santa Catarina; Head of ENT-HNS, University Hospital of the Federal University of Santa Catarina). Department of Otorhinolaryngology and Head and Neck Surgery - University Hospital - Federal University of Santa Catarina (UFSC)

**Keywords:** hearing aids, hearing loss, patient satisfaction, rehabilitation

## Abstract

Some individuals with hearing loss choose to be fitted with hearing aids. Compliance is significantly affected by how satisfied patients are with their hearing aids. Patient satisfaction can be assessed through questionnaires and scales.

**Objective:**

To assess the degree of satisfaction of patients fitted with hearing aids.

**Method:**

Scale “Satisfaction With Amplification in Daily Life” (SADL) was applied to 180 patients fitted with hearing aids; results were categorized based on the mean values observed for global satisfaction scores and scores attained on each subscale. Patients were interviewed for additional information.

**Results:**

Mean global score was 5.6; 48.9% of the subjects were very satisfied, 47.2% were satisfied, and 3.9% were dissatisfied. The mean score on subscale Positive Effects was 5.6; the mean score for Service and Cost was 6.2; for Negative Factors the mean score was 4.9; and the mean score on subscale Personal Image was 5.8. Of the patients fitted with in-the-ear hearing aids, 83.3% were very satisfied. Fifteen percent of the individuals were dissatisfied with their Personal Image. Sensorineural and profound hearing loss patients were less satisfied, with 5.4% and 50.0% of the subjects revealing dissatisfaction with their hearing aids.

**Conclusion:**

Patients were generally very satisfied with their hearing aids. Satisfaction rates were higher among patients fitted with in-the-ear hearing aids. Dissatisfaction was higher in subscale Personal Image. Lesser degrees of satisfaction were seen in patients with sensorineural and profound hearing loss.

## INTRODUCTION

Hearing loss or hypacusis is characterized by reductions on the ability to hear and/or detect sounds, and may arise in various stages on an individual's life^1^, ^2^.

Many conditions in which hearing loss occurs concurrently may be addressed through clinical treatment and/or surgery. However, many types of hearing loss cannot be treated using these approaches. In these cases, hearing aids become an important tool in the auditory training and/or rehabilitation of the involved subjects^3^.

Hearing aids have been significantly improved with the development of technology in the area. The contributions yielded from such progress refer mainly to air conduction aids and include component miniaturization, enhancements in components such as amplifiers, and the introduction of new algorithms to reduce noise and intensify speech detection^4^. Different types of devices are currently available, and since 2004 the Brazilian Health System has provided hearing aids to patients at specialized outpatient clinics^5^.

Objective and subjective methods can be used to assess the benefits of hearing aids to their users. Subjective methods resort to scales to assess patient satisfaction levels. One of the tools used with this purpose is the Satisfaction With Amplification in Daily Life scale - SADL - designed by Cox & Alexander^6^. The SADL scale yields a global score (GS) of satisfaction with hearing aids and specific scores to assess satisfaction in the following subscales: positive effect (PE), service and cost (SC), negative features (NF), and personal image (PI)^6^. The SADL scale was developed and validated by Cox & Alexander^6^ based on a sample of 257 subjects with a mean age of 72 years from a war veteran care center in the United States, a community hearing and speech care center, and a private audiology clinic^7^. The scale was translated into Brazilian Portuguese and given the title *Satisfação com o Aparelho Auditivo em sua Vida Diária*^8^. According to a study by Danieli et al.^9^, the scale is an effective means to assess the satisfaction of patients fitted with hearing aids provided by the Brazilian Health System. Adapted versions of the SADL have been used by other Brazilian authors^10^, ^11^, ^12^, ^13^, ^14^.

A study carried out with hearing aid users from the army health system revealed considerable levels of satisfaction with the devices, despite the low scores on subscale negative features, particularly in what concerns using a telephone^10^. Lower satisfaction levels in this subscale have been reported in other studies based on the SADL scale^11^, ^12^, ^13^. These reports support the findings of a previous study: difficulty using a telephone is one of the most relevant items in low hearing aid satisfaction scores^15^.

The army health system study also indicated that the subjects with fewer complaints of auditory involvement (intolerance to intense sounds and tinnitus) and without bilateral sensorineural hearing loss had better outcomes with their hearing aids^10^. The global scores and subscale scores reported by the authors were consistent with the scores published in the original study by Cox & Alexander^6^, ^10^.

Other Brazilian studies using the SADL scale found higher global satisfaction scores than the standardized values reported in the original study^11^, ^12^, ^13^, ^14^. It is worth mentioning that one of these studies was carried out with patients with severe and profound hearing loss^11^. Another study suggested that the type of device had statistically affected satisfaction scores. Subscales PE, PI, and GS were significantly correlated, and correlations were stronger for patients wearing in-the-ear devices when compared to behind-the-ear hearing aids^14^.

According to Arawaka et al.^16^, in Brazil and in other countries such as the United States, the rates of dissatisfaction with hearing aids have been as high as 47%, with 18% of the subjects giving up on auditory rehabilitation. Thus, to these authors, successful hearing aid fitting is correlated with how satisfied the patient is with the outcome provided by the device^16^.

The satisfaction of subjects fitted with hearing aids is affected by the benefit yielded by the device. It is also associated with user expectations, cost of treatment, psychological aspects, issues faced during the rehabilitation process, and communication difficulties that remain despite the use of amplification^6^.

Therefore, this study aimed to assess the degree of satisfaction of subjects fitted with hearing aids seen at a high complexity service.

## METHOD

This cross-sectional contemporary cohort study was carried out at a high complexity service between August 18, 2011 and August 2, 2012. The study was approved by the Ethics Committee for Research with Human Beings of the institution and was given permit 2140/2011.

The study included individuals fitted with hearing aids at the hospital's auditory care unit.

The following enrollment criteria were applied: subjects had to be 14 or older, have hearing loss of any kind or degree in at least one ear, had to be wearing their hearing aids for at least 30 days, and had to agree to join the study and sign an Informed Consent Term.

Patients with ages under 14 years, individuals fitted with hearing aids at other care centers, and subjects who could not or refused to join the study were excluded.

The following variables were analyzed: age, gender, education, marital status, type of hearing loss in each ear, degree of hearing loss, type of hearing aids, time since using the current hearing aids, number of hours per day with hearing aids on, presence of comorbidities, and level of satisfaction with the hearing aids based on the SADL scale (Annex 1)^8^.

In order to prevent interpretation errors or biased results, each individual was given a copy of the scale to follow the items with the help of a caretaker while the questions were asked orally by the researcher. Patient charts were used as reference to collect subject personal data.

The SADL scale contains 15 items and assesses the satisfaction of hearing aid users through a global score and a set of specific scores related to the following subscales: PE (items 1, 3, 5, 6, 9, and 10), SC (items 12, 14, and 15), NF (items 2, 7, and 11), and PI (items 4, 8, and 13). Participants answered the questions by picking one of the following possible answers: not at all; a little; somewhat; medium; considerably; greatly; tremendously. In 11 items, ‘tremendously' indicates maximum satisfaction and is assigned a score of seven, while ‘not at all' means maximum dissatisfaction and is given a score of one (items 1, 3, 5, 6, 8, 9, 10, 11, 12, 14, 15). However, the other items in the scale are reversed, and ‘tremendously' suggests maximum dissatisfaction and is given a score of one, while ‘not at all' indicates maximum satisfaction and is assigned a score of seven^17^.

The scores in the four subscales were calculated based on the mean value of the scores attained in each component item. The subjects in this study were given their hearing aids free of charge, and thus item 14 was not considered. Therefore, subscale SC was limited to two items (12 and 15). Additionally, when subjects claimed they could hear well on the phone without hearing aids, question 11 was suppressed from subscale NF, thus limiting it to two items (2 and 7). The satisfaction GS was calculated from the mean value of the scores attained in the 14 or 13 - for patients who could hear well on the phone without hearing aids - items of the SADL scale.

After the SADL scores of our sample were calculated, they were compared to the standard scores as shown in Table 1^6^, ^18^. The reference values were used to determine the profile of the individuals in each subscale and in the satisfaction global score. Subjects were deemed to be ‘dissatisfied' when their scores were under the 20^th^ percentile according to the standard, ‘very satisfied' when their scores were above the 80^th^ percentile, and ‘satisfied' when their scores were between the 20^th^ and 80^th^ percentiles.

**Table 1:** Mean global and subscale scores and their respective standard deviations for the 20^th^ and 80^th^ percentiles of the SADL scale according to the paper published by Cox and Alexander (1999)^6^.

Score	Mean	Standard deviation	20^th^ percentile	80^th^ percentile
Global	4.9	0.8	4.3	5.6
Service and cost	4.7	1.2	4.5*	6.5*
Positive effect	4.9	1.3	3.8	6.1
Negative features	3.6	1.4	2.3	5.0
Personal image	5.6	1.4	5.0	6.7

* Subjects were given their hearing aids free of charge.

The Brazilian Portuguese version of the SADL scale contains four additional items. Yet, this study considered only the time since wearing hearing aids and the number of hours per day with hearing aids on^8^.

The descriptive analysis of the data was carried out on Microsoft Excel 2010^®^.

## RESULTS

This study included 180 individuals, 72 (40%) males and 108 (60%) females. Subject mean age was 65.1 ± 15.5 years, ranging from 14 to 94. In terms of education, 23 (12.8%) patients were illiterate, 115 (63.9%) went to elementary school, 28 (15.6%) attended middle school, and 14 (7.8%) had higher education degrees. When marital status was considered, 100 (55.6%) subjects were married or were in a steady union and 80 (44.4%) were single, separated, or widows. Fifteen (8.3%) did not have comorbidities and/or tinnitus, and 165 (91.7%) had. Eight (4.4%) patients had unilateral hearing loss and 172 (95.6%) had bilateral deafness, adding to a total of 352 ears with hearing loss.

The mean global and subscale scores are shown in Table 2.

**Table 2:** SADL global and subscale scores of the patients included in this study.

SADL subscale	Mean	Minimum	Maximum	Standard deviation
Positive effect	5.6	2.2	7.0	0.9
Service and cost	6.2	2.0	7.0	1.0
Negative features	4.9	1.0	7.0	1.3
Personal image	5.8	2.7	7.0	1.1
Global score	5.6	3.4	6.9	0.7

According to patient global scores, 88 (48.9%) subjects were very satisfied, 85 (47.2%) were satisfied, and seven (3.9%) were dissatisfied.

**Figure 1:**
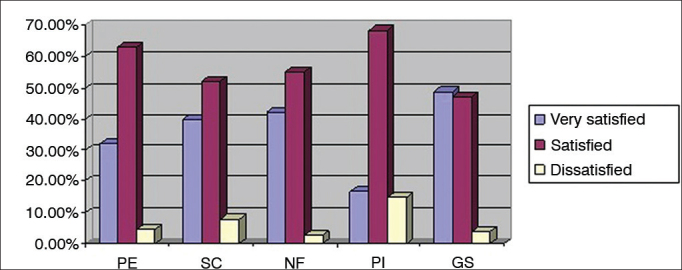
Percent distribution of very satisfied, satisfied, and dissatisfied patients in each subscale and satisfaction global scores in the SADL scale. PE: Positive effect; SC: Service and cost; NF: Negative features; PI: Personal image; GS: Global score.

The levels of satisfaction for each SADL subscale are presented in Figure 1. Patients were categorized as very satisfied, satisfied, or dissatisfied in each SADL subscale and in terms of their global scores.

**Figure 2:**
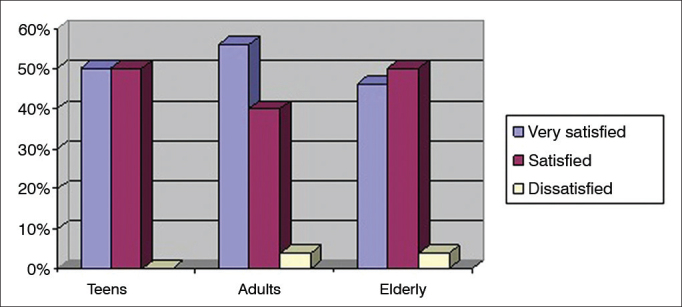
Percent distribution of patient ages per level of global satisfaction.

In order to assess the correlation between age and level of satisfaction, patients were divided into three groups: teens (12 to 17 years of age), adults (18 to 59 years of age), and elderly (60 years of age and above). The teen group had two (1.1%) subjects, the adult group had 50 individuals (27.8%), and the elderly group had 128 (71.1%) members. Figure 2 shows the percentages of individuals in each age group categorized based on levels of satisfaction.

**Figure 3:**
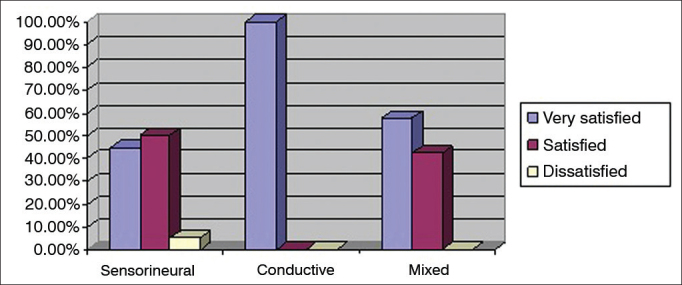
Percent distribution of hearing loss types per level of global satisfaction.

Each ear was considered individually in the assessment of degree of satisfaction based on type of hearing loss. Eight (2.3%) ears had conductive hearing loss, 261 (74.1%) had sensorineural hearing loss, and 83 (23.6%) had mixed hearing loss. Figure 3 shows the levels of satisfaction categorized as a percentage based on the types of hearing loss.

**Figure 4:**
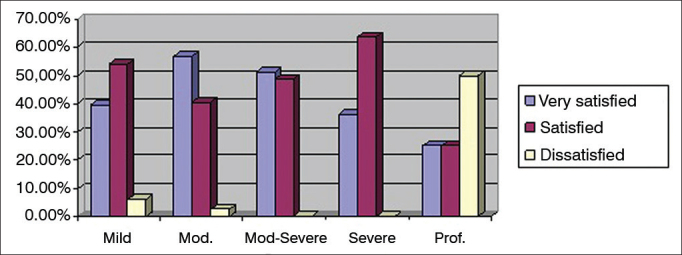
Percent distribution of hearing loss degrees per level of global satisfaction. Mod.: Moderate; Mod-Severe: Moderate-severe; Prof.: Profound.

The better ear of the patients with bilateral hearing loss was considered in the assessment of level of satisfaction based on hearing loss type. Forty-eight (26.7%) patients had mild hearing loss; 74 (41.1%) had moderate hearing loss; 43 (23.9%) had moderate to severe hearing loss; 11 (6.1%) had severe hearing loss; and four (2.2%) had profound hearing loss. Figure 4 shows the categorization of level of satisfaction as a function of degree of hearing loss as a percentage.

**Figure 5:**
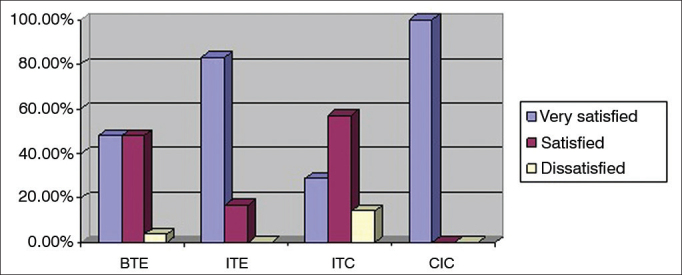
Percent distribution of hearing aid types per level of global satisfaction. BTE: Behind the ear, ITE: In the ear; ITC: In the canal; CIC: Completely in the canal.

The 330 devices fitted were air conduction hearing aids. Three-hundred and eight (93.3%) were behind-the-ear (BTE) devices, six (1.8%) were in-the-ear (ITE) aids, 14 (4.2%) were intracanal (ITC) devices, and two (0.6%) were completely-in-the-canal (CIC) hearing aids. Figure 5 shows the categorization of level of satisfaction as a function of type of hearing aids on each ear.

**Figure 6:**
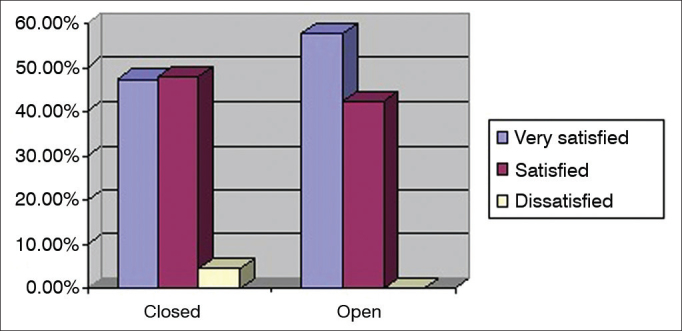
Percent distribution of hearing aid fitting type per level of global satisfaction.

Lastly, the level of satisfaction with the type of fitting was analyzed. Three-hundred and four (92.1%) hearing aids had closed fittings and 26 (7.9%) had open fittings. Figure 6 shows the the categorization of level of satisfaction as a function of type of fitting.

## DISCUSSION

Female subjects accounted for 60% of the population included in this study. Predominance of female over male individuals was also reported by Lessa et al.^11^, in a study on hearing aid users with severe and profound hearing loss, and by Soares et al.^12^. The opposite, i.e., predominance of male over female subjects, was described by Carvalho^13^ and Farias & Russo^14^, albeit to a lesser degree in the latter. Veiga et al.^10^ found equal numbers of male and female subjects.

Carvalho^13^ studied strictly elderly individuals and reported a mean age of 72.2 years. The other studies mentioned above included patients aged 18 and older. The patients described by Veiga et al.^10^ had a mean age of 72 years; Soares et al.^12^ reported a mean age 58.7 years in their group; Farias e Russo^14^ reported a mean age of 58.2 years; and Lessa et al.^11^ described a population with a mean age of 52.3 years. The mean age of the patients included in this study is in agreement with the literature. Nonetheless, differently from other studies, it included patients aged 14 years and older. Future studies should consider including younger subjects, as attempted in this study despite the limited number of enrolled teenagers. Patients in this age range have their own peculiarities in terms of psychological development and prevalent diseases, and may respond differently when confronted with the use of hearing aids.

Patient mean satisfaction GS was 5.6 ± 0.7, i.e., the subjects were satisfied with their devices. The breakdown of the global score indicated that 48.9% of the patients were very satisfied, 47.2% were satisfied, and 3.9% were dissatisfied. Subscale PI stood out from the pack as 15% of the individuals were dissatisfied, although the mean score suggested they were satisfied. Cox & Alexander^6^ also reported that, for some people, their self-image while wearing hearing aids and the impression they cause upon others were extremely important, though many users seemed not to worry about it. Likewise, Carvalho^13^ reported high satisfaction rates with hearing aids in all SADL domains, but a greater number of dissatisfied individuals on subscale PI. It should be noted that such outcomes may be related to behind-the-ear aids, the device type worn by all patients in the sample, which may have become a confounding factor. In contrast, Veiga et al.^10^ concluded that patients generally did not correlate wearing hearing aids with portraying an image of disability.

Russo et al.^19^ looked into the meaning of hearing loss and having to wear hearing aids for elderly individuals. The authors found that both matters were strongly linked to the stigma of hearing loss, thus affecting compliance rates of patients in this age range. Another study with elderly individuals ranked stigma as the least important factor for users of hearing aids, but as the biggest concern for patients unwilling to wear hearing aids, males in particular^20^. Erler & Garstecki^21^ analyzed the stigma related to hearing loss and to the use of hearing aids for female patients and found the negative perceptions related to hearing aids were correlated with age. Younger female subjects had stronger negative perceptions related to the stigma of hearing loss and wearing hearing aids than older women.

Therefore, the appearance of the devices and the stigma connected to wearing hearing aids and having hearing loss adversely affect a considerable portion of the patients. It is important to stress that this study looked into the factors connected to lower satisfaction ratings of individuals who chose to use hearing aids. We do not know why patients with hearing loss decide not to wear hearing aids. Awareness building campaigns are needed to inform patients of the potential benefits of wearing hearing aids and to educate the population in general on the principles of equality and tolerance.

Considering the factors related to differences in levels of satisfaction, patients with sensorineural hearing loss were more dissatisfied than individuals with mixed or conductive hearing loss. The mean GS of patients with sensorineural hearing loss was 5.5 ± 0.7, suggesting they were satisfied when compared to standard values, but 5.4% were dissatisfied against none with other types of hearing loss. Veiga et al.^10^ reported higher chances of satisfaction among patients without bilateral hearing loss with purely sensorineural components.

Cochlear function is normal in subjects with conductive hearing loss. Therefore, the quality of hearing is expected to be better in these patients as they use hearing aids than in subjects with sensorineural hearing loss, in whom some degree of discrimination involvement and recruitment may be present^22^. Additionally, many subjects with sensorineural hearing loss have limited hearing range^23^. However, although the processed component of sensorineural hearing loss cannot be repaired, amplified volume increases audibility and reduces the effort needed to comprehend sound in daily living activities^24^. Therefore, in agreement with this study, subjects with conductive hearing loss and most patients with sensorineural hearing loss are expected to be highly satisfied with wearing hearing aids. The latter group is also benefitted, despite more significant limitations.

Patients with profound hearing loss also had lower satisfaction global scores. Their mean GS was 4.6 ± 0.9, which indicates they were satisfied, however less than the study's general population. Additionally, 50% of the subjects with profound hearing loss were dissatisfied. This finding may be explained by the presence of less residual hearing to take advantage of amplification. It should be noted that all patients with profound hearing loss in this study wore BTE aids, possibly a confounding factor. Differently from this study and from Veiga et al.^10^, Soares et al.^12^, and Carvalho^13^, Lessa et al.^11^ reported a high degree of satisfaction among severe and profound hearing loss patients, with higher mean global scores and no dissatisfied subjects. The authors concluded that the patients had a lot of communication difficulties prior to hearing aid fitting and that the devices provided them with some degree of assistance, thus enabling satisfaction to prevail.

High satisfaction global scores were seen among patients fitted with ITE aids, and a well above the average mean GS of 6.4 ± 0.5. In this group of patients, 83.3% were very satisfied, 16.7% were satisfied, and none were dissatisfied. It is important to mention that all users of ITE aids in this study had moderate to severe hearing loss. Farias & Russo^14^ also reported relatively higher levels of satisfaction among patients fitted with ITE aids in subscales PE, PI, and in the global score. Additionally, Fialho et al.^25^ studied elderly subjects fitted with hearing aids and found patients were less willing to wear BTE aids for cosmetic reasons.

To Wrobel & Gabard^24^, ITE aids are preferred by patients because they are cosmetically more attractive than BTE aids, allow better location of sound sources, and are made up of only one component. The authors also listed cosmetics as an advantage for ITC and CIC aids, but higher satisfaction global scores among users of ITC or CIC aids were not seen in our study. In regards to CIC devices, the low number of users may have been a contributing factor. The absence of reports of higher satisfaction scores among users of ITC aids may be related to the fact that they are suitable only for cases of mild to moderate hearing loss, to the fragility of the hearing aids, to the difficulties they pose to patients with impaired hand dexterity, to the limited possibilities of settings adjustments due to the small size of the device, or to the device's limited ventilation options^24^. Thus, the satisfaction with ITE aids may be higher because they are more discrete than BTE aids and easier to handle and adjust than ITC aids, as they are bigger in size.

In general terms, the population enrolled in this study was satisfied with the hearing aids during the 30 days of the fitting process, regardless of type and degree of hearing loss. More studies on patient satisfaction with longer observation periods may bring new useful data to the assessment of hearing aids.

## CONCLUSION

This study revealed patients were highly satisfied with their hearing aids, as most subjects were very satisfied or satisfied and only a few were dissatisfied with their devices. Dissatisfaction was more noticeable on subscale personal image, and was a relevant finding among patients with profound hearing loss and, to a lesser degree, among subjects with sensorineural hearing loss.

In-the-ear hearing aids were correlated with higher satisfaction scores, as most users were very satisfied and none were dissatisfied with their devices.

## Figures and Tables

**Figure:**